# A Comparison of the Operations for Vesico-Vaginal Fistula

**Published:** 1877-04

**Authors:** 


A Comparison of the Operations for Vesico-Vaginal Fistula of N. Bozeman, M.D., New York, and Prof. Gustav. Simon Heidelberg, in a pamphlet published by A. D. Bernays, M.D., of St. Louis, Mo., is before us. This translation is published by the translator in order to correct some errors on the part of Dr. Bozeman in regard to the matter.

The principal point noticed in this comparison is the more perfect simplicity of Simon's operation, with a showing of as good statistical results, if not better than Bozeman's. While Simon places the patient in cocygeo-dorsal position for operating, Bozeman operates in the genu-pectoral. Simon uses silk sutures, and very indifferent after-treatment, while Bozeman applies silver and button sutures and careful after-treatment.

In fact, it seems to us about "six one way and a half dozen the other," and the comparison narrows down to a question of the time required to operate.

